# Screening of fetal alcohol syndrome using the T-ACE questionnaire in semi-rural areas around Lubumbashi: lessons learned

**DOI:** 10.11604/pamj.2014.19.264.4348

**Published:** 2014-11-11

**Authors:** Toni Kasole Lubala, Nina Lubala, Augustin Mulangu Mutombo, Olivier Mukuku, Oscar Numbi Luboya

**Affiliations:** 1 Faculty of Medecine, University of Lubumbashi, BP 1825, Democratic Republic of Congo

**Keywords:** Screening, fetal alcohol syndrome, T-ACE questionnaire, Lubumbashi

## To the editors of the Pan African Medical Journal

African women consumption of alcohol is influenced by a number of beliefs and sociocultural considerations. In Lubumbashi in the D.R.Congo for example, the belief that alcohol reduces sympathetic signs of pregnancy during the first trimester is very common; this belief however exposes unborn children to ethanol toxicity at a very early stage of the pregnancy. Hence, there is an urgent need to educate the future mothers (and the population) on the risks associated with alcohol consumption (even in small amount) during pregnancy.

In Lubumbashi (southern D.R.Congo) very little is known about the bad effect and the toxicity of alcohol during pregnancy. In addition to that, alcohol consumption by African women is influenced by strong beliefs and cultural considerations which expose unborn babies to irreversible toxic effects of Ethanol. Moderate alcohol consumption of alcohol being more frequent than excessive consumption among pregnant women, its possible bad effect on unborn children have an important impact in public health [1]. Our study on alcohol consumption conducted on 300 women randomly chosen in the three principal maternity clinics in semi-rural areas around Lubumbashi showed that 37% had risky alcohol consumption during their pregnancy. The women concerned by the study were relatively young and so susceptible to have other pregnancies (average age: 28 years, extreme age: 16-41 years).

These results gathered by using the T-ACE (T: Tolerance, A: Annoyance, C: Cut down, E: Eye opener) questionnaire [2] made in place for the screening of alcohol consumption of pregnant women might have been influenced by the tendency by these women to deny or to minimize their consumption of alcohol out of embarrassment [1, 2]. Nevertheless, these results pointed out a serious problem of public health. 98% of the women interrogated in our study have admitted not knowing the teratogen effect of Ethanol. In addition to that, 30% think that alcohol, beer in particular reduces sympathetic signs of pregnancy in the first trimester; this widespread belief unfortunately exposes foetuses to the toxicity of alcohol during the crucial phase of embryogenesis. 2% of the mothers strongly believed that fermented “munkoyo” (traditional beer) contribute to the good development of the foetus; note also that in Lubumbashi (DRCongo) giving birth to a macrosomic baby is considered ideal and this misconception exposes the unborn child to the toxicity of Ethanol [3–5].

In this contest, our concern was particularly on the moderate but on going consumption of alcohol which causes minor mental retardation, persistent physical and neurodevelopmental abnormalities like hyperkinesis or decrease attention span [3, 4]. Many longitudinal studies on pre-school children have showed that antenatal alcohol exposure can have long term effect on the intellectual development of children and on their performances at school [1, 4]. Impaired intellectual capacity and a deficit in neurobehavioral and social skill were observed. It is clear that foetal alcohol exposure has a long term bad effect on the neurodevelopment of children. We believe that authorised publicity of alcoholic beverages should be regulated in view of improving the knowledge of pregnant women. All containers should have clarified notices or images with a message like this: “alcohol consumption even in small amount during pregnancy can have serious consequences on the child health”. In addition, mothers education can be intensify in the mother and children protection activities programs (prenatal consultations and preschool consultation) already in place in the primary health care plan of the Democratic Republic of Congo.

## Conclusion

In Lubumbashi (south of DRCongo), mostly in semi-rural areas we have noticed an important rate of alcohol consumption among pregnant women due greatly to misleading socio-cultural beliefs. There is without any doubts an urgent need to educate the mothers and the community on the dangers of alcohol consumption (even in relatively small amount) during any stage of pregnancy. In addition, we believe that breweries can be used in the effort to teach pregnant women and the community on the real danger of alcohol consumption during pregnancy.
